# Mutational Analysis of EGFR and Related Signaling Pathway Genes in Lung Adenocarcinomas Identifies a Novel Somatic Kinase Domain Mutation in *FGFR4*

**DOI:** 10.1371/journal.pone.0000426

**Published:** 2007-05-09

**Authors:** Jenifer L. Marks, Michael D. McLellan, Maureen F. Zakowski, Alex E. Lash, Yumi Kasai, Stephen Broderick, Inderpal S. Sarkaria, DuyKhanh Pham, Bhuvanesh Singh, Tracie L. Miner, Ginger A. Fewell, Lucinda L. Fulton, Elaine R. Mardis, Richard K. Wilson, Mark G. Kris, Valerie W. Rusch, Harold Varmus, William Pao

**Affiliations:** 1 Human Oncology and Pathogenesis Program, Memorial Sloan-Kettering Cancer Center, New York, New York, United States of America; 2 Genome Sequencing Center, Washington University School of Medicine, St. Louis, Missouri, United States of America; 3 Department of Pathology, Memorial Sloan-Kettering Cancer Center, New York, New York, United States of America; 4 Computational Biology Program, Memorial Sloan-Kettering Cancer Center, New York, New York, United States of America; 5 Department of Surgery, Memorial Sloan-Kettering Cancer Center, New York, New York, United States of America; 6 Thoracic Oncology Service, Department of Medicine, Memorial Sloan-Kettering Cancer Center, New York, New York, United States of America; 7 Program in Cancer Biology and Genetics, Memorial Sloan-Kettering Cancer Center; 8 Department of Medicine, Weill Medical College of Cornell University, New York, New York, United States of America; Deutsches Krebsforschungszentrum, Germany

## Abstract

**Background:**

Fifty percent of lung adenocarcinomas harbor somatic mutations in six genes that encode proteins in the EGFR signaling pathway, i.e., *EGFR, HER2/ERBB2, HER4/ERBB4, PIK3CA, BRAF*, and *KRAS*. We performed mutational profiling of a large cohort of lung adenocarcinomas to uncover other potential somatic mutations in genes of this signaling pathway that could contribute to lung tumorigenesis.

**Methodology/Principal Findings:**

We analyzed genomic DNA from a total of 261 resected, clinically annotated non-small cell lung cancer (NSCLC) specimens. The coding sequences of 39 genes were screened for somatic mutations via high-throughput dideoxynucleotide sequencing of PCR-amplified gene products. Mutations were considered to be somatic only if they were found in an independent tumor-derived PCR product but not in matched normal tissue. Sequencing of 9MB of tumor sequence identified 239 putative genetic variants. We further examined 22 variants found in RAS family genes and 135 variants localized to exons encoding the kinase domain of respective proteins. We identified a total of 37 non-synonymous somatic mutations; 36 were found collectively in *EGFR, KRAS, BRAF*, and *PIK3CA*. One somatic mutation was a previously unreported mutation in the kinase domain (exon 16) of *FGFR4* (Glu681Lys), identified in 1 of 158 tumors. The *FGFR4* mutation is analogous to a reported tumor-specific somatic mutation in *ERBB2* and is located in the same exon as a previously reported kinase domain mutation in *FGFR4* (Pro712Thr) in a lung adenocarcinoma cell line.

**Conclusions/Significance:**

This study is one of the first comprehensive mutational analyses of major genes in a specific signaling pathway in a sizeable cohort of lung adenocarcinomas. Our results suggest the majority of gain-of-function mutations within kinase genes in the EGFR signaling pathway have already been identified. Our findings also implicate FGFR4 in the pathogenesis of a subset of lung adenocarcinomas.

## Introduction

Lung cancer is the leading cause of cancer-related death in the United States and worldwide [1]. Despite recent advances in the treatment of lung cancer, the overall 5-year survival in the United States remains only 15%, highlighting the need for novel treatment strategies.

Lung cancers are currently classified into two major groups depending on histology: small cell lung cancer and non-small cell lung cancer (NSCLC). The latter is comprised of three different subtypes: adenocarcinoma, squamous cell carcinoma, and large cell carcinoma. The incidence of the adenocarcinoma subtype has been rising and now accounts for >50% of all cases of lung cancer [2]. Standard treatment for metastatic lung cancer involves empiric cytotoxic chemotherapy.

In order to develop specific therapies based upon the genetic makeup of individual NSCLC tumors, we (the Lung Cancer Oncogenome Group at Memorial Sloan-Kettering Cancer Center (MSKCC)) and others have sought to define clinically relevant molecular subsets of lung cancer. For example, we and others have shown that tumors highly sensitive to epidermal growth factor receptor (*EGFR*) tyrosine kinase inhibitors (i.e. gefitinib or erlotinib) often contain dominant mutations in exons which encode a portion of the tyrosine kinase (TK) domain of *EGFR* [3]–[5]. Conversely, tumors with somatic mutations in *KRAS*, which encodes a GTPase downstream of EGFR, are resistant to therapy with these drugs [6]–[8]. Furthermore, about half of tumors with acquired resistance to these drugs display a second-site mutation in *EGFR* (Thr790Met) [9], [10]. Taken together, these data suggest that molecularly defined subgroups of lung cancer indeed exist and can be used to predict sensitivity and resistance to gefitinib and erlotinib. Clinicians in the future may be able to prescribe additional targeted therapies for patients with NSCLC based upon specific molecular characteristics.

At least six EGFR signaling pathway genes have been found to be mutated in NSCLC. While *EGFR* and *KRAS* mutations are detected in ∼10% and 20% of NSCLCs, respectively, somatic mutations have also been identified in *HER2/ERBB2* (∼2%; exons 19 and 20) [11], [12] and *HER4* (∼2%, exons 20, 23) [13], the lipid kinase *PIK3CA* (∼4%; exon 9) [14], and the serine/threonine kinase *BRAF* (∼2%; exons 11 and 15) [15]–[17]. Most of these alterations have been found to be gain-of-function mutations. Except for *PIK3CA* mutations [18], [19], mutations in one of the other five genes are rarely found to be accompanied by a mutation in any of the remaining four, suggesting that they may have functionally equivalent roles in lung tumorigenesis [20]. All of these mutations are predominantly found in tumors with adenocarcinoma histology.

To uncover other potential gain-of-function somatic mutations that could have biological and clinical relevance in lung cancer, we performed mutational profiling of a large cohort of lung tumors, mostly adenocarcinomas. Because multiple genes that encode proteins in the EGFR signaling pathway have been found to be mutated in lung adenocarcinomas, we specifically sought to identify potential gain-of-function mutations in gene families in this pathway, i.e. in *ERBB1-4, PIK3CA, AKT1-3, FRAP1, RPS6K1-2, RAS (K-, N-*, and *H-*), *RAF (A-, B-, C-*), *MAP2K1-2*, and *MAPK-1-3*. We extended our studies to include other members of the MAP2K and MAPK gene families. We also examined *FGFR1-4*, because overexpression of FGF ligands in mouse lung epithelia leads to alveolar type II cell hyperplasia and adenomas [21]–[23]. All 39 genes have been reported to be expressed in mammalian lung tissues.

## Methods

### Tissue procurement

Resected tumor and matched normal adjacent lung specimens were obtained with patients' consent from the Memorial Sloan-Kettering Cancer Center (MSKCC) lung cancer tissue bank via a protocol approved by the Institutional Review Board (protocol #92-055). At the time of resection, samples were snap-frozen in the operating room in liquid nitrogen and then stored at minus 80°C until the time of use. Specimens were reviewed by a single pathologist (MFZ) for ≥70% tumor content and for histological verification. Clinical information was obtained from existing institutional databases. Some data regarding the mutation status of *EGFR* was previously reported [5].

### Mutational profiling

Tumors selected for analyses were enriched for lung adenocarcinomas but were otherwise randomly selected, based upon availability of tissue. Squamous cell carcinomas were included to fill-in otherwise empty plate wells. No large cell carcinomas were studied.

DNA was extracted from tumors using a kit (DNeasy, Qiagen) or standard phenol extraction. Whole genome amplification (WGA) was performed by Qiagen. High-throughput (96-well plate) bidirectional dideoxynucleotide sequencing of PCR-amplified gene products was performed at the Genome Sequencing Center (Washington University in St. Louis) as per standard protocol (http://genome.wustl.edu/activity/med_seq/protocols.cgi). The primer list can be found at: http://genome.wustl.edu/platforms.cgi?id = 7.

PolyPhred [24] and PolyScan [25] software were used to generate an initial “automated” report of sequence variations. Tumor sequences were compared against reference sequences listed in the NCBI (RefSeq) database for each respective gene (**see Supplemental Table S1**). After visual inspection of the individual forward and reverse chromatograms for confirmation of non-synonymous sequence variations and insertions or deletions (including duplications), a “manual review” list of potential nucleotide changes was produced. Synonymous variants and those with corresponding dbSNP (www.ncbi.nlm.nih.gov/projects/SNP/) entries were also excluded.

### Mutation verification

Putative kinase domain mutations listed in the manual report were subsequently verified at MSKCC by bidirectional sequence analysis of a separate individual PCR product. Variants were deemed somatic if they were found to be absent in matched normal tissue. Primers were designed to detect each individual mutation, using each respective reference sequence and Vector NTI (**Supplemental Tables S1** **and S2**). All PCR reactions were performed with HotStarTaq Master Mix Kit (Qiagen, Valencia, California), using standard conditions (95°C×15 min; 95°C×30 s, 60°C×30 s, 72°C×60 s, for 36 cycles, then 72°C for 5 minutes, 50 µl reactions). PCR products were purified with a MultiScreen Resist vacuum manifold and PCR_96_ Cleanup Plates (Millipore). Sequencing reactions were performed using Applied Biosystems Version 3.1 Big Dye Terminator chemistry and analyzed on an Applied Biosystems 3730 Sequencer.

### Development of “Mutagrator” – a mutation interpretation tool for tyrosine kinases

To support the interpretation of putative kinase domain mutations, we created a prototype mutation interpretation tool for tyrosine kinases (TKs), called “Mutagrator”, located at http://cbio.mskcc.org/∼lash/mutagrator/ (freely available to the research community). Mutagrator is a software program which takes curated mutation data from the literature and displays it in the context of a master protein (chosen by the user) and a protein-registered TK multiple domain alignment. In order to create the multiple alignment, we first retrieved 108 human TK gene records from EntrezGene by querying for domain cd00192 [26]. We then extracted TK domains from all 168 protein isoforms corresponding to these genes from Entrez Protein [27], aligned the domains using the ClustalW program [28], and added additional feature information, including ATP binding residues, activation loop, catalytic loop and substrate binding site boundaries from Conserved Domain Database (CDD) [29]. All input and output files are available on the Mutagrator website. Currently, curated mutation data is ingested from the Catalogue of Somatic Mutations in Cancer (COSMIC), which was created and is maintained by the Sanger Institute [30]. Collected data includes mutation (amino acid change and position), mutation type (point, insertion, deletion, complex), involved gene, tissue type, cancer type and published source. The version of the database used in this study (v20) consisted of about 30,000 individual mutations in about 1,300 genes, and corresponding to about 3,300 distinct mutations. From these data, Mutagrator produced interlinked, static HTML webpages of two types: master protein pages (for each protein in the TK domain alignment with mutations), and detailed mutation pages (for each protein residue position).

## Results

We screened coding sequences from 39 genes for mutations in genomic DNA from a total of 261 resected, clinically annotated non-small cell lung cancer (NSCLC) specimens. 90% of tumors were adenocarcinomas, and 10% were squamous cell carcinomas. Clinical characteristics of examined tumors are listed in **Supplemental Table S3**, and the exonic coverage of genes is listed in **Supplemental Table S1**.

**Figure 1:**
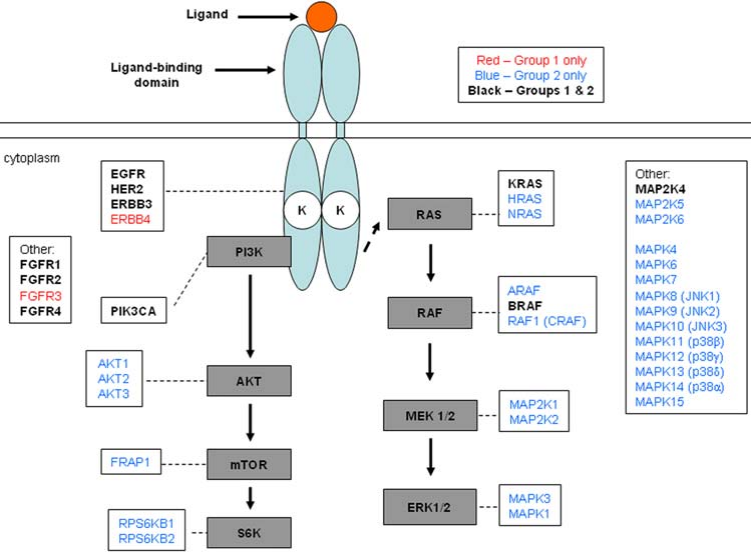
Genes sequenced in this study. The schematic diagram depicts the EGFR signaling pathway. Genes listed in red were sequenced only in genomic DNAs from 217 tumors (“Group 1”). Genes listed in blue were sequenced only in WGA-treated DNA tumor samples (“Group 2”). Genes in black were sequenced in both groups. Gene nomenclature is as reported in GenBank as of December 2006. See Supplemental Table S3 for clinical characteristics of all tumors sequenced.

Due to logistical reasons, the mutational analysis was performed in two partially overlapping groups. We first examined genomic DNAs from 217 tumors for mutations in a set of core genes previously reported to harbor mutations in NSCLC, i.e. in *EGFR, HER2, HER4, KRAS, PIK3CA*, and *BRAF* **(** **Figure 1** **)**. We also profiled *HER3, MAP2K4*, and *FGFR1-4* **(** **Figure 1** **)**. We then examined 93 WGA-treated DNA tumor samples for mutations in EGFR pathway genes and a set of exploratory genes **(** **Figure 1** **)**. Ten genes were sequenced in both groups **(** **Figure 1** **)** to maximize the number of tumors sequenced for the core genes. Eighty percent of the sequence reads in the WGA-treated specimens had a Phred quality score of at least 20 (data not shown), suggesting that most base-calling had an accuracy of 99% [31].

**Figure 2:**
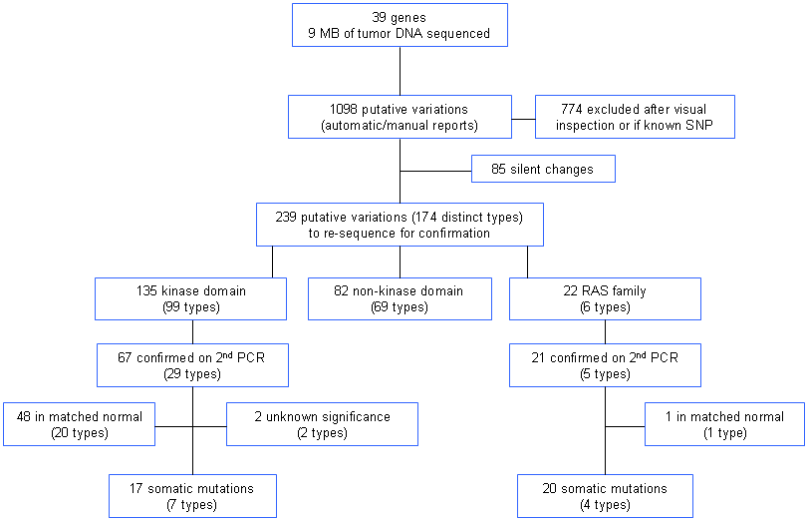
Schematic of overall results. A putative variation was defined as a sequence variation compared to a reference sequence in GenBank. After visual inspection and exclusion of known SNPs and silent changes, there were 239 tumor sequences with a variation representing 174 distinct types of variations. The sequence variations were further divided into three groups: 135 variations (99 distinct types) within exons encoding the kinase domains of respective genes, 82 variations (69 types) in exons encoding areas outside the kinase domains of respective kinase genes, and 22 variations (6 types) in RAS family genes. Non-synonymous variations confirmed by sequence analysis of a 2^nd^ PCR were either somatic mutations or variants found in matched normal tissue (listed in Supplemental Table S4). The significance of two novel variants, *ERBB2* (exon 20, Arg784Cys) and *MAPK6* (exon 4, Val262Ile), is unclear, because we could not determine if the variants were also found in DNA from corresponding normal tissue.

Automatic and manual sequence analyses (see methods) identified 239 putative non-synonymous sequence variations, comprised of 174 different types of variants that differed from published sequences **(** **Figure 2** **, and Supplemental Table S1)**. To focus our efforts, we concentrated on further examining the 22 variants (6 types) found in 3 RAS family genes and the 135 variants (99 types) found within exons encoding kinase domains of kinases. The 82 non-kinase domain variants (69 distinct types) have not yet been examined, although none occur at a frequency higher than 2%.

We confirmed 21 of the sequence variations in the *RAS* family. 20 were somatic (all in codons 12 or 13 of exon 2 of *KRAS*), while one in *HRAS* was found in matched normal DNA **(Supplemental Table S4)**. The prevalence of *KRAS* mutations in our cohort of lung adenocarcinomas was 12% (20/173). All confirmed somatic mutations were found in adenocarcinomas except for a Gly12Asp mutation in *KRAS* in a squamous cell carcinoma **(** **Table 1** **)**.

**Table 1:** Clinical characteristics of patients whose tumors contained a somatic mutation.

Tumor	Mutation	Age	Gender	Histology	Smoking	Stage
*EGFR* mutant						
20t^1^	exon 19 del	62	F	AWBF	Never	IB
230t^1^	exon 19 del	71	F	ADENO	Never	IA
261t	exon 19 del	68	M	AWBF	Former (>15 pk yr)	IA
303t	exon 19 del	76	M	AWBF	Former (>15 pk yr)	IB
317t	exon 19 del	77	F	AWBF	Former (≤15 pk yr)	IA
433t	exon 19 del	81	F	AWBF	Never	IB
428t	exon 20 dup	61	M	AWBF	Former (≤15 pk yr)	IA
5t^1^	Leu858Arg	53	F	ADENO	Former (≤15 pk yr)	IA
65t^1^	Leu858Arg	85	M	AWBF	Former (≤15 pk yr)	IA
98t^1^	Leu858Arg	64	F	AWBF	Former (≤15 pk yr)	IA
134t^1^	Leu858Arg	68	M	ADENO	Former (≤15 pk yr)	IA
250t	Leu858Arg	45	M	AWBF	Never	IIIA
251t	Leu858Arg	67	F	AWBF	Former (>15 pk yr)	IA
*KRAS* mutant						
12t	Gly12Val	52	F	ADENO	Current	IB
70t	Gly12Val	58	M	ADENO	Current	IIIA
86t	Gly12Val	58	M	ADENO	Former (>15 pk yr)	IIB
109t	Gly12Val	78	F	AWBF	Never	IB
110t	Gly12Val	47	M	ADENO	Current	IIB
404t	Gly12Val	70	F	ADENO	Current	IIIB
6t	Gly12Cys	75	M	AWBF	Former (>15 pk yr)	IA
29t	Gly12Cys	78	F	ADENO	Former (>15 pk yr)	IB
64t	Gly12Cys	74	M	ADENO	Former (>15 pk yr)	IIB
87t	Gly12Cys	70	M	AWBF	Former (>15 pk yr)	IA
290t	Gly12Cys	75	F	ADENO	Former (>15 pk yr)	IIIB
357t	Gly12Cys	63	F	ADENO	Former (>15 pk yr)	IIA
376t^2^	Gly12Cys	67	F	ADENO	Former (>15 pk yr)	IB
439t	Gly12Cys	59	M	AWBF	Former (>15 pk yr)	IA
1t	Gly12Asp	75	F	AWBF	Former (>15 pk yr)	IV
37t	Gly12Asp	67	F	AWBF	Former (>15 pk yr)	IB
L29t	Gly12Asp	76	F	SCC	Never	IIB
67t	Gly12Asp	69	M	AWBF	Former (>15 pk yr)	IA
68t	Gly12Asp	80	M	AWBF	Former (>15 pk yr)	IIIA
69t	Gly13Cys	66	F	AWBF	Former (>15 pk yr)	IA
*PIK3CA* mutant						
376t^2^	Glu545Lys	67	F	AWBF	Former (>15 pk yr)	IB
421t	Glu545Lys	80	F	ADENO	Former (>15 pk yr)	IA
*BRAF* mutant						
408t	Val600Glu	64	M	AWBF	Former (>15 pk yr)	IB
*FGFR4* mutant						
410t	Glu681Lys	66	F	ADENO	Current	IIIB

Smoking history is defined as never smokers (<100 lifetime cigarettes), former smokers (quit ≥1 year prior to diagnosis), or current (quit <1 year prior to diagnosis. Former smokers were further defined as having smoked ≤15 pack years (number of packs of cigarettes smoked per day multiplied by the number of years the person has smoked) or >15 pack years.

1 Mutation previously reported (5).

2 Tumor contained both a *KRAS* and *PIK3CA* mutation. Abbreviations: ADENO, adenocarcinoma; AWBF, adenocarcinoma with bronchioalveolar features (38); SCC, squamous cell carcinoma; dup, duplication; del, deletion. AWBF is equivalent to the WHO classification: adenocarcinoma, mixed subtype, with BAC component (39).

67 of the 135 kinase domain sequence variations were confirmed by analysis of sequence tracings from an independent PCR isolate. 48 variants were also found in corresponding normal samples **(Supplemental Table S4)**. Two were of uncertain significance, because we were unable to obtain a PCR product from DNA from matched normal tissue **(Supplemental Table S4)**. Of the remaining 17 confirmed non-synonymous somatic variants, 16 were found in genes known to be mutated in NSCLC, i.e. *EGFR, BRAF*, and *PIK3CA* **(Supplemental Table S4)**. The prevalence of *EGFR, BRAF*, and *PIK3CA* mutations in lung adenocarcinomas was 6 (13/234), <1 (1/156), and 2% (2/132), respectively. Clinical characteristics of all tumors containing somatic mutations can be found in **Table 1**. One *PIK3CA* mutation was found in a tumor that also contained a *KRAS* mutation. No other tumor had more than one somatic mutation **(** **Table 2** **)**.

**Table 2:** Mutations observed in *EGFR, KRAS, BRAF*, and *FGFR4* in lung adenocarcinomas.

# Samples	*EGFR*	*KRAS*	*BRAF*	*FGFR4*
13^1^	X	wt	wt	wt
20^2^	wt	X	wt	wt
1	wt	wt	X	wt
1	wt	wt	wt	X

Except for one *KRAS* mutant tumor which also contained a *PIK3CA* mutation, no other tumor with a mutation in one of these genes had a mutation in the other 3 genes. X denotes a mutation.

1 5 EGFR mutations were previously reported (5).

2 One *KRAS* mutation was found in a squamous cell carcinoma. Abbreviations: wt, wild-type.

**Figure 3:**
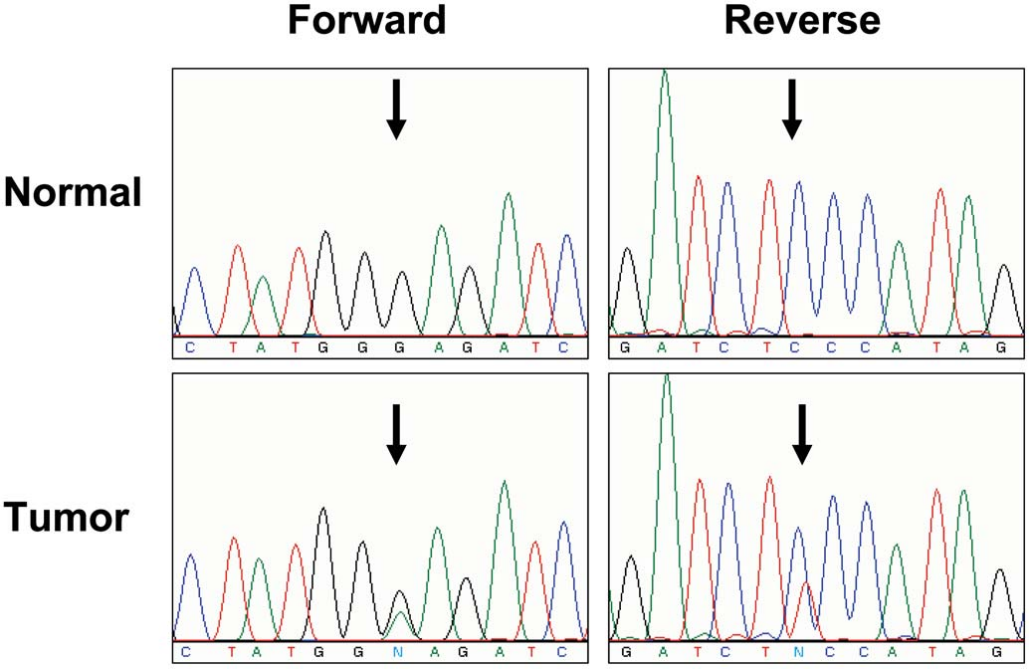
Analysis of FGFR4. Forward/reverse sequencing chromatograms for the mutation identified in exon 16 of *FGFR4* in tumor and matched normal samples. The nucleotide change is c.2041G>A, that would lead to substitution of lysine for glutamic acid at position 681.

**Figure 4:**
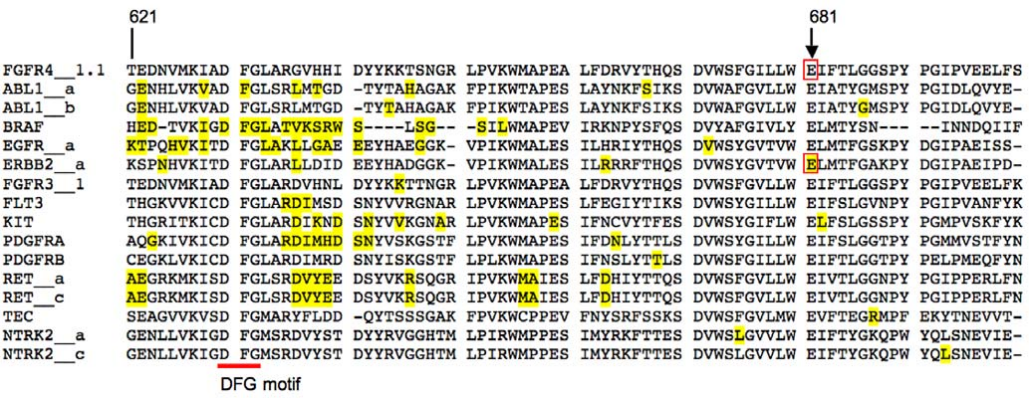
Amino acid alignment of the FGFR4 kinase domain with other tyrosine kinase domains found to be altered in human cancers. The DFG motif found in all kinases is underlined. The glutamic acid residue at position 681 in FGFR4 (boxed) is highly conserved amongst the various kinases. Amino acids affected by mutations and reported in the COSMIC (Catalogue of Somatic Mutations in Cancer) database appear in yellow. The analogous Glu914 residue in ERBB2 (boxed) has been found to be mutated in a glioblastoma. Figure adapted from a screenshot of the “Mutagrator” bioinformatics tool developed for this study. The previously reported Pro712Thr mutation in FGFR4 was also identified by the Mutagrator tool but is not shown. See methods for more details.

In one lung adenocarcinoma specimen from a current smoker, we found a somatic heterozygous G to A mutation at nucleotide position 2041 in exon 16 of *FGFR4* **(** **Figure 3** **)**. This mutation would lead to substitution of lysine for glutamic acid at position 681 (Glu681Lys), 51 amino acids downstream of the highly conserved DFG motif found in all protein kinases **(** **Figure 4** **)**. Using our “Mutagrator tool” **(** **Figure 4**; see methods **)**, we determined that an analogous mutation has been reported in a glioblastoma in *ERBB2* (Glu914Lys) [11]. Moreover, the glutamic acid at position 681 is highly conserved among various kinases **(** **Figure 4** **)**. The biological significance of the lung *FGFR4* mutation remains to be determined experimentally. In total, this mutation was found in 1 of 158 tumors. We did not identify any other somatic mutations in this tumor **(** **Table 2** **)**.

## Discussion

We report a comprehensive sequencing study of major genes in a specific signaling pathway in a sizeable cohort of lung adenocarcinoma tumor specimens. Previous large-scale mutational profiling studies of lung cancer have examined either only the exons encoding the activation loops of receptor tyrosine kinase (RTK) genes (47 of 58 RTK genes) in 119 primary NSCLCs, of which 70 (59%) were lung adenocarcinomas [4], or the coding sequences of 518 protein kinases in a relatively limited number of samples, i.e. 26 primary lung neoplasms (7 adenocarcinomas) and seven cancer cell lines (6 adenocarcinomas) [32]. Here, we examined a total of 261 tumor samples, predominantly adenocarcinomas, specifically for genetic alterations in genes encoding major signaling proteins in the EGFR signaling pathway. We also determined the status of a select set of other genes potentially relevant to lung tumorigenesis.

Most of the somatic mutations we found have been reported, including mutations in *EGFR, KRAS, BRAF,* and *PIK3CA* [3]–[5], [11]–[17]. The relative distribution of these mutations in our lung adenocarcinomas matches that observed by others. The frequency of *EGFR* and *KRAS* mutations was slightly lower than other published series, possibly because the mutation detection software that we used went through various stages of development during this project [25]. We did not identify any somatic mutations in *HER2* or *HER4*. However, one of two variants of uncertain significance (due to inability to PCR amplify a gene product from matched normal DNA) was located in the kinase domain of *HER2* (Arg784Cys) and has not been previously reported.

**Figure 5:**
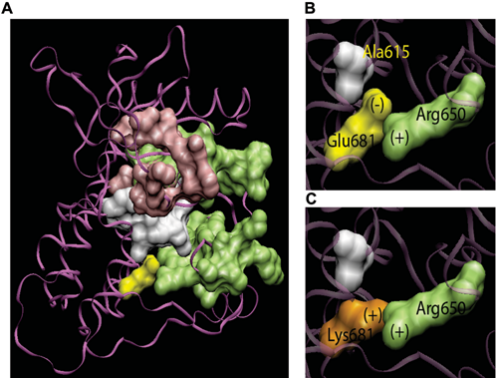
Structural modeling of the FGFR4 Glu681Lys amino acid substitution. A. The FGFR4 WT and E681K mutant structures are predicted using the PROTINFO software (38) provided by the (PS)2 server (National Chiao Tung University, Taiwan). These predictions are based on crystallographic structure for FGFR1 tyrosine kinase domain (PDB accession 1FGK) (33), as no FGFR4 structure is available, and visualized using VMD (39). FGFR4 Glu681 (yellow), ATP binding site (pink), activation loop (green) and catalytic loop (white). Glu681 (yellow) is nestled between the TK activation and catalytic loops. B. 3D close-up of the surfaces of Glu681 (yellow), Arg650 (green) in the activation loop, and Ala615 (white) in the catalytic loop. Since Glu681 is strongly negatively charged and Arg650 is strongly positively charged, ionic bonding between these two closely juxtaposed residues may be assumed. C. 3D close-up of the surfaces of mutated Lys681 (orange), Arg650 and Ala615. The glutamic acid to lysine substitution at position 681 could structurally and functionally alter the kinase domain by flipping the charge of residue 681 and disrupting ionic bonds with neighboring residues, particularly the closely juxtaposed Arg650.

We did find a novel mutation (Glu681Lys) in the kinase domain (exon 16) of *FGFR4* in 1 of 158 tumors. This mutation is analogous to the previously reported Glu914Lys kinase domain mutation in *ERBB2* found in a glioblastoma [11]. Glu681 is highly conserved region among various kinases, downstream of the DFG motif. Based on the crystal structure of the related family member FGFR1 tyrosine kinase domain (PDB accession 1FGK) [33], the analogous residue (Glu692) appears in close proximity to Ala626 in the TK catalytic loop and Arg661 in the TK activation loop. Since Glu692 is strongly positively charged and Arg661 is strongly negatively charged, the close spatial proximity of these two residues would likely lead to a strong ionic bond and therefore may be functionally important. Extrapolating back to FGFR4, we propose that the Glu681Lys mutation may alter the functional properties of the TK catalytic domain by reversing the charge of residue 681, potentially disrupting an ionic bond with residue Arg650, and thereby disrupting normal function of FGFR4 **(** **Figure 5** **)**.

FGFR4 is a monomeric receptor protein tyrosine kinase possessing three immunoglobulin-like domains in the extracellular region. The protein is one of four high-affinity receptors for multiple members of the FGF family of ligands that evoke angiogenic, mitogenic, and differentiation responses in cells [34]. Such ligands, when overexpressed in mouse lung epithelia, stimulate alveolar type II cell hyperplasia and adenoma formation [21]–[23]. Interestingly, Davies et al have reported that a lung adenocarcinoma cell line also harbors a non-synonymous mutation in exon 16 of *FGFR4* – Pro672Thr [32]. [The Davies et al paper referenced *FGFR4* transcript variant 2; we referenced variant 1, so the equivalent mutation would be Pro712Thr.] Collectively, these data suggest a role for *FGFR4* mutations in a subset of lung adenocarcinomas. The Sanger group also found two other somatic mutations in genes that encode the related family members, *FGFR1* and *FGFR2*, in lung cancer specimens. The described *FGFR1* and *FGFR2* mutations occur outside the kinase domain, but in identical positions to activating germline mutations known to predispose to skeletal dysplasias. Other *FGFR* gene alterations have also been reported in human cancers, although rarely in exons encoding the kinase domain (reviewed in [34]). We plan to characterize the functional consequences of the two reported *FGFR4* mutations and determine their prevalence in independent lung and other tumor specimen banks.

This study has some potential limitations. First, we examined only 39 genes. We did not sequence all related gene family members such as *RPS6KA1-6, MAP2K3*, and *MAP2K7*. This study also did not seek potential mutations in genes encoding adaptor proteins or phosphatases that might affect the ERBB signaling pathway. Second, WGA could have skewed the results by selectively amplifying DNA from normal rather than tumor tissue. However, evaluation of data from multiple assays has established that base-calling discrepancies between amplified and unamplified samples are minimal and not significantly different than that observed after re-sequencing non-amplified samples [35], [36]. Consistent with this, in all cases where we found an *EGFR* or *KRAS* mutation in the original non-WGA-treated sample, we also detected the same mutation in the corresponding WGA-treated sample (n = 14; data not shown). Finally, in this initial study, we restricted our verification studies to non-synonymous variants in the exons encoding kinase domains, in view of the clinical significance of known somatic mutations in kinase domains. The 69 types of non-kinase domain sequence variations we identified are currently undergoing confirmation. Nevertheless, the prevalence thus far of non-synonymous somatic mutations per megabase of tumor sequenced in this study was 4.1 (37 total mutations/9Mb). This rate is slightly higher than that found by others in a mutational analysis of ∼13,000 genes in 11 colorectal and 11 breast cancers [37].

This study represents an early step towards an understanding of the lung cancer oncogenome. Our results suggest that the majority of gain-of-function mutations within kinase genes in the EGFR signaling pathway may have been identified. We await results from the NCI/NHGRI-sponsored “technical demonstration project” – a pilot project for The Cancer Genome Atlas initiative, in which approximately 200 highly-curated lung adenocarcinomas are being analyzed for chromosomal gains and losses simultaneously with mutational profiling of about 1000 genes thought to be relevant to lung tumorigenesis. Efforts such as these should contribute towards the identification of the full spectrum of somatic mutations found in lung adenocarcinomas.

## Supporting Information

Table S1 Gene, GenBank accession number, and exonic coverage of genes sequenced in this study.
(0.06 MB DOC)

Table S2 List of primers used to verify putative variants.
(0.15 MB DOC)

Table S3 Clinical characteristics of patients whose tumors were analyzed. Group 1 was used for sequencing the “core” genes. Group 2 was used for sequencing the “exploratory” genes. Some tumors and genes overlapped between the two groups. Smoking history is defined as never smokers (<100 lifetime cigarettes), former smokers (quit ≥1 year prior to diagnosis), or current (quit <1 year prior to diagnosis). See text and Figure 1 for more detail. 1Adeno includes adenocarcinoma with bronchioalveolar features (n = 79, n = 27 for Group 1 and 2, respectively). Abbreviations: Adeno, adenocarcinoma; SCC, squamous cell carcinoma.
(0.04 MB DOC)

Table S4 List of variants verified. Group headings correspond to groups in bottom row of Figure 2. Variants found in normal tissue did not have an existing entry in dbSNP. 1A total of 5 EGFR mutations (exon 19 del, n = 1: exon 21 L858R, n = 4) have been previously reported (5). 2Variants with high frequency were not verified in all samples. If a variant was also found in DNA from five matched normals, no further samples were verified. Abbreviations: del, deletion; dup, duplication.
(0.08 MB DOC)

## References

[1] Jemal A, Siegel R, Ward E, Murray T, Xu J (2006). Cancer statistics, 2006.. CA Cancer J Clin.

[2] Gabrielson E (2006). Worldwide trends in lung cancer pathology.. Respirology.

[3] Lynch TJ, Bell DW, Sordella R, Gurubhagavatula S, Okimoto RA (2004). Activating mutations in the epidermal growth factor receptor underlying responsiveness of non-small-cell lung cancer to gefitinib.. N Engl J Med.

[4] Paez JG, Janne PA, Lee JC, Tracy S, Greulich H (2004). *EGFR* mutations in lung cancer: correlation with clinical response to gefitinib therapy.. Science.

[5] Pao W, Miller V, Zakowski M, Doherty J, Politi K (2004). EGF receptor gene mutations are common in lung cancers from “never smokers” and are associated with sensitivity of tumors to gefitinib and erlotinib.. Proc Natl Acad Sci USA.

[6] Pao W, Wang TY, Riely GJ, Miller VA, Pan Q (2005). *KRAS* mutations and primary resistance of lung adenocarcinomas to gefitinib or erlotinib.. PLoS Medicine.

[7] Han S-W, Kim T-Y, Hwang PG, Jeong S, Kim J (2005). Predictive and prognostic impact of epidermal growth factor receptor mutation in non-small-cell lung cancer patients treated with gefitinib.. J Clin Oncol.

[8] Giaccone G, Gallegos Ruiz M, Le Chevalier T, Thatcher N, Smit E (2006). Erlotinib for frontline treatment of advanced non-small cell lung cancer: a phase II study.. Clin Cancer Res.

[9] Pao W, Miller VA, Politi KA, Riely GJ, Somwar R (2005). Acquired resistance of lung adenocarcinomas to gefitinib or erlotinib is associated with a second mutation in the EGFR kinase domain.. PLoS Medicine.

[10] Kobayashi S, Boggon TJ, Dayaram T, Janne PA, Kocher O (2005). *EGFR* mutation and resistance of non-small-cell lung cancer to gefitinib.. New Engl J Med.

[11] Stephens P, Hunter C, Bignell G, Edkins S, Davies H (2004). Lung cancer: intragenic ERBB2 kinase mutations in tumours.. Nature.

[12] Shigematsu H, Takahashi T, Nomura M, Majmudar K, Suzuki M (2005). Somatic mutations of the *HER2* kinase domain in lung adenocarcinomas.. Cancer Res.

[13] Soung YH, Lee JW, Kim SY, Wang YP, Jo KH (2006). Somatic mutations of the ERBB4 kinase domain in human cancers.. Int J Cancer.

[14] Samuels Y, Wang Z, Bardelli A, Silliman N, Ptak J (2004). High frequency of mutations of the PIK3CA gene in human cancers.. Science.

[15] Naoki K, Chen TH, Richards WG, Sugarbaker DJ, Meyerson M (2002). Missense mutations of the BRAF gene in human lung adenocarcinoma.. Cancer Res.

[16] Brose MS, Volpe P, Feldman M, Kumar M, Rishi I (2002). BRAF and RAS mutations in human lung cancer and melanoma.. Cancer Res.

[17] Davies H, Bignell G, Cox C, Stephens P, Edkins S (2002). Mutations of the BRAF gene in human cancer.. Nature.

[18] Endoh H, Yatabe Y, Kosaka T, Kuwano H, Mitsudomi T (2006). *PTEN* and *PIK3CA* expression is associated with prolonged survival after gefitinib treatment in *EGFR*-mutated lung cancer patients.. J Thor Oncol.

[19] Kawano O, Sasaki H, Endo K, Suzuki E, Haneda H (2006). PIK3CA mutation status in Japanese lung cancer patients.. Lung Cancer.

[20] Shigematsu H, Nomura M, Suzuki M, Wistuba II, Fujisawa T (2005). Gene mutation differences in lung cancers arising in never and ever smokers.. Proc AACR.

[21] Clark J, Tichelaar J, Wert S, Itoh N, Perl A (2001). FGF-10 disrupts lung morphogenesis and causes pulmonary adenomas in vivo.. Am J Physiol Cell Mol Physiol.

[22] Tichelaar J, Lu W, Whitsett J (2000). Conditional expression of fibroblast growth factor-7 in the developing and mature lung.. J Biol Chem.

[23] Zhao B, Chua S, Burcin M, Reynolds S, Stripp B (2001). Phenotypic consequences of lung-specific inducible expression of FGF-3.. Proc Natl Acad Sci U S A.

[24] Nickerson DA, Tobe VO, Taylor SL (1997). PolyPhred: automating the detection and genotyping of single nucleotide substitutions using fluorescence-based resequencing.. Nucleic Acids Research.

[25] Chen K, McLellan MD, Ding L, Wendl MC, Kasai Y (in press). PolyScan: an automatic indel and SNP detection approach to the analysis of human re-sequencing data.. Genome Res.

[26] Maglott D, Ostell J, Pruitt KD, Tatusova T (2006). Entrez Gene: gene-centered information at NCBI.. Nucleic Acids Res.

[27] Wheeler DL, Barrett T, Benson DA, Bryant SH, Canese K (2006). Database resources of the National Center for Biotechnology Information.. Nucleic Acids Res.

[28] Chenna R, Sugawara H, Koike T, Lopez R, Gibson TJ (2003). Multiple sequence alignment with the Clustal series of programs.. Nucleic Acids Res.

[29] Marchler-Bauer A, Anderson JB, Derbyshire MK, Deweese-Scott C, Gonzales NR (2006). CDD: a conserved domain database for interactive domain family analysis.. Nucleic Acids Res.

[30] Forbes S, Clements J, Dawson E, Bamford S, Webb T (2006). Cosmic 2005.. Br J Cancer.

[31] Ewing B, Hillier L, Wendl MC, Green P (1998). Base-calling of automated sequencer traces using phred. I. Accuracy assessment.. Genome Research.

[32] Davies H, Hunter C, Smith R, Stephens P, Greenman C (2005). Somatic mutations of the protein kinase gene family in human lung cancer.. Cancer Res.

[33] Mohammadi M, Schlessinger J, Hubbard SR (1996). Structure of the FGF receptor tyrosine kinase domain reveals a novel autoinhibitory mechanism.. Cell.

[34] Eswarakumar VP, Lax I, Schlessinger J (2005). Cellular signaling by fibroblast growth factor receptors.. Cytokine Growth Factor Rev.

[35] Bredel M, Bredel C, Juric D, Kim Y, Vogel H (2005). Amplification of whole tumor genomes and gene-by-gene mapping of genomic aberrations from limited sources of fresh-frozen and paraffin-embedded DNA.. J Mol Diagn.

[36] Paez JG, Lin M, Beroukhim R, Lee JC, Zhao X (2004). Genome coverage and sequence fidelity of phi29 polymerase-based multiple strand displacement whole genome amplification.. Nucleic Acids Research.

[37] Sjoblom T, Jones S, Wood LD, Parsons DW, Lin J (2006). The Consensus Coding Sequences of Human Breast and Colorectal Cancers. Science..

[38] Ebright MI, Zakowski MF, Martin J, Venkatraman ES, Miller VA (2002). Clinical pattern and pathologic stage but not histologic features predict outcome for bronchioloalveolar carcinoma.. Ann Thorac Surg.

[39] Brambilla E, Travis WD, Colby TV, Corrin B, Shimosato Y (2001). The new World Health Organization classification of lung tumours.. Eur Respir J.

