# Supplementary material for: Mutational Analysis of EGFR and Related Signaling Pathway Genes in Lung Adenocarcinomas Identifies a Novel Somatic Kinase Domain Mutation in FGFR4
Source: PLoS One. 2007 May 9;2(5):e426. doi: 10.1371/journal.pone.0000426 (PMC1855985; doi:10.1371/journal.pone.0000426)
Supplement: Table S1 — Gene, GenBank accession number, and exonic coverage of genes sequenced in this study. (0.06 MB DOC) [file pone.0000426.s001.doc]

| **Gene** | **GenBank accession** | **Exons Sequenced** |
| --- | --- | --- |
| *AKT1* | NM_005163.2 | 2-14 |
| *AKT2* | NM_001626.2 | 2-14 |
| *AKT3* | NM_181690.1 | 1-13 |
| *ARAF* | NM_001654.1 | 2-10,14,16 |
| *BRAF* | NM_004333.3 | 9,11,15 |
| *EGFR* | NM_005228.3 | 2-28 |
| *ERBB2* | NM_001005862.1 | 1-27 |
| *ERBB3* | NM_001982.2 | 1-28 |
| *ERBB4* | NM_005235.1 | 2-22, 24-28 |
| *FGFR1* | NM_023108 | 1-19 |
| *FGFR2* | NM_000141.3 | 2-19 |
| *FGFR3* | NM_000142.2 | 2-17 |
| *FGFR4* | NM_213647.1 | 1-18 |
| *FRAP1* | NM_004958.2 | 2-58 |
| *HRAS* | NM_176795.2 | 3-7 |
| *KRAS* | NM_033360.2 | 2-6 |
| *MAP2K1* | NM_002755.2 | 2,3,5-11 |
| *MAP2K2* | NM_030662.2 | 3,4, 7-11 |
| *MAP2K4* | NM_003010.2 | 2-11 |
| *MAP2K5* | NM_145160.1 | 1-23 |
| *MAP2K6* | NM_002758.3 | 2-11 |
| *MAPK1* | NM_002745.4 | 2-9 |
| *MAPK3* | NM_001040056.1 | 2-8 |
| *MAPK4* | NM_002747.3 | 2-8 |
| *MAPK6* | NM_002748.2 | 2-6 |
| *MAPK7* | NM_139034.1 | 2-9 |
| *MAPK8* | NM_002750.2 | 1-11 |
| *MAPK9* | NM_139070.1 | 1-11 |
| *MAPK10* | NM_002753.2 | 1-13 |
| *MAPK11* | NM_002751.5 | 5-12 |
| *MAPK12* | NM_002969.3 | 3-11 |
| *MAPK13* | NM_002754.3 | 3-12 |
| *MAPK14* | NM_001315.1 | 1-12 |
| *MAPK15* | NM_139021.2 | 1-11 |
| *NRAS* | NM_002524.2 | 1-7 |
| *PIK3CA* | NM_006218.2 | 9, 20 |
| *RAF1* | NM_002880.2 | 1-17 |
| *RPS6KB1* | NM_003161.2 | 1-9, 13-15 |
| *RPS6KB2* | NM_003952.2 | 1-15 |

**Supplemental Table S1.** **Gene, GenBank accession number, and exonic coverage of genes sequenced in this study.**
