# Supplementary material for: Mutational Analysis of EGFR and Related Signaling Pathway Genes in Lung Adenocarcinomas Identifies a Novel Somatic Kinase Domain Mutation in FGFR4
Source: PLoS One. 2007 May 9;2(5):e426. doi: 10.1371/journal.pone.0000426 (PMC1855985; doi:10.1371/journal.pone.0000426)
Supplement: Table S2 — List of primers used to verify putative variants. (0.15 MB DOC) [file pone.0000426.s002.doc]

| **GENE** | **Exon** | **Forward primer** | |  | |  |  | **Reverse primer** | |
| --- | --- | --- | --- | --- | --- | --- | --- | --- | --- |
| ***AKT1*** | 10 | GTGTACCGGGACCTCAAGGT | | | |  |  | ATTGCGTGTGCTCAGGACGT | |
|  | 11 | CAATGCTGTGTCCTCTCTGT | | | |  |  | AGTGTAGTCTGGGAGGTGCC | |
|  | 12 | GGGCCCTACATCACAGGAGGAA | | | | |  | ACCTGGGAAATCTGGCGAGCGTG | |
| ***AKT2*** | 8 | AGGGCAGGGTGTGTGTGTGCAGAA | | | | |  | TCCCGCCCCACCCTAAAGAA | |
|  | 10 | GGCATCTTTCCCTGGAAGGA | | | |  |  | GGAAACACACAGGTCTGGGG | |
|  | 12 | AGGGCCTGCCTGCTCCGAAA | | | |  |  | AACCAAGGTCACCACGAGTG | |
| ***AKT3*** |  | none |  | |  |  |  |  |  |
| ***ARAF*** | 16 | GCTGGGGCTGTTGGGATGCC | | | |  |  | ATTGGCTGGTGGGCAAGGCT | |
| ***BRAF*** | 11 | CTGTATCCCTCTCAGGCATA | | | |  |  | GCGAACAGTGAATATTTCCT | |
|  | 15 | TCATAATGCTTGCTCTGATAGGA | | | | |  | GGCCAAAAATTTAATCAGTGG | |
| ***EGFR*** | 19 | GTCACAGCCCCCAGCAATATCA | | | | |  | GGATGTGGAGATGAGCAGGG | |
|  | 21 | TAACGTTCGCCAGCCATAAG | | | |  |  | CGAGCTCACCCAGAATGTC | |
| ***ERBB2*** | 20 | CACCGTGCCCGGCCTAATCTTT | | | | |  | TGCCCAGCAAGAGTCCCCAT | |
| ***ERBB4*** | 23 | TCTTTTACTACTGGTATAGTGC | | | |  |  | CAGATTGAGTAATCTCTGCT | |
| ***FGFR1*** | 10 | TGCCTCTGCCATTGTTGGGA | | | |  |  | TGAACCTTCACCGCCCAGAA | |
|  | 12 | GGAAAAGCAGCCCCTCGACA | | | |  |  | TGAGAGAGGCCTTGGGACTG | |
| ***FGFR2*** | 12 | ACATATTTCCTTTTTGTTCTGGCGG | | | | |  | TCAAAAGAACGGGAATCGGG | |
|  | 9 | CATGCTTTAGTAAGCCGCTG | | | |  |  | TTCAACATCTTCACGGCCAC | |
| ***FGFR3*** | 13 | AGGTGTGGGTGGAGTAGGCT | | | |  |  | AGGCGTCCTACTGGCATGAC | |
| ***FGFR4*** | 10 | GCATCCCCCACCTCACATGT | | | |  |  | ACACAGCTCAGCGCACCTGT | |
|  | 13 | AAACCTGCGGGAGTTCCTGC | | | |  |  | ACATTGAATGCCACAGGCCT | |
|  | 16 | TGGGTCATGTCTGTGGGGTC | | | |  |  | ACTGGAAAGTGGGGTCGAGG | |
| ***FRAP1*** |  | none |  | |  |  |  |  |  |
| ***HRAS*** | 6 | CCAGGCAAGGCTTGATCCCA | | | |  |  | ATGTCCTGAGCTTGTGCTGGGC | |
| ***KRAS*** | 2 | GTGTGACATGTTCTAATATAGTCA | | | | |  | GAATGGTCCTGCACCAGTAA | |
| ***MAP2K1*** | 2 | GAGGCCTTGCAGAAGAAGCT | | | |  |  | GGTCCCCAGGCTTCTAAGTA | |
|  | 3 | ACAAGACTATATCTTTCATCCC | | | |  |  | AGACCAAAGATTAGGCCAGA | |
|  | 7 | ACCCAGGGGTCCAAGTTAGG | | | |  |  | CCTCCCCTCCAACAGTCCAA | |
|  | 8 | GATCCATGCCCAACCCCTTG | | | |  |  | GGCCACCTGGGTGTTTTCTT | |
| ***MAP2K2*** | 9 | AGTGAGGGGTAGAGAGGGCT | | | |  |  | TGCACTGGGATTCTGGATGG | |
| ***MAP2K4*** | 3 | AAAACTTCAAAAACCTGGAGGTC | | | | |  | GGTATTACTGCACCATGAAATT | |
|  | 4 | AATTTTTAGTCTCGTAACGG | | | |  |  | CTTCATTTGTCCCAATTAGA | |
|  | 6 | GCAGAGGACTACACGGGATA | | | |  |  | CCGTTTCACCAATTAGGAGA | |
|  | 9 | GTTTAGATTTTTTTGTGGCCCTTC | | | | |  | ACCAATGCTGCTAAGACCAA | |
| ***MAP2K5*** | 12 | TTTATACTACACATCCAATGCATGC | | | | |  | CCAACTGGGATTGAAGAACA | |
|  | 19 | GCTGTTGTTGATTAATCTTG | | | |  |  | TACAACACAACTTGCTCCAA | |
| ***MAP2K6*** |  | none |  | |  |  |  |  |  |
| ***MAPK1*** | 4 | TTTTCTCTCTTAGATCTGTGAC | | | |  |  | GACTTACGGCTTACTGCAAT | |
|  | 5 | AAGTTCAGAGTCCCATCAGC | | | |  |  | TGTGGTTCAGCTGGTCAAGA | |
| ***MAPK3*** | 4 | GCGTGACTGCTTAGGCTGCA | | | |  |  | AGGTAGCTCCAGGGCTTCCT | |
|  | 6 | CTGTTCCCAGAAAGAAAGAG | | | |  |  | ACACCCACCCTCATGTCTCT | |
|  | 7 | CGCTGGAGGAGACAGGCAAA | | | |  |  | ACTGCTGCTGGGGACTGGCC | |
| ***MAPK4*** | 2 | TGAGAAGGGTGACTGCATCG | | | |  |  | CTTGATCTCTCGGAGCGCGT | |
|  | 2.1 | CACTGACCTGCAGGGTGAGCTGTT | | | | |  | AAGATGTTGGCGGGCTTCAGGTCC | |
|  | 5 | ATCGACTTTCTGGAGAAGAT | | | |  |  | AATAGAGATGGGAGGACAGG | |
| ***MAPK6*** | 2 | CTCCCTTTTTGAAAGATCTT | | | |  |  | TGTAAACACTGTTCAGTTCC | |
|  | 2.1 | GGCTTGGTTTTTTCTGCTGTAGA | | | | |  | ATGAGGATCCATGATCCGTG | |
|  | 4 | GTTTGTTTTTTAACCTCAG | | | |  |  | GAGTCACAATACCTTCTCGA | |
|  | 5 | GTACAATTAAGATGCTCATTTCCTG | | | | |  | TGGCTCATCCATTGGAAAAG | |
| ***MAPK7*** | 2 | CTCTGAAGGAGGAAGACGGC | | | |  |  | CTTTCCTTCCCGGCTGTCTG | |
|  | 3 | GAGGTGTTGATAGGGGCTGA | | | |  |  | TTGAATTCGCCATAGGGCAC | |
|  | 4 | GGGCTGCATCTTTGGTGAGA | | | |  |  | TGCGACCCAGCAGTGATAGG | |
|  | 4.1 | CCACCCTTTCCTGGCCAAGT | | | |  |  | TCAATGGTGTCAGGTGCAGG | |
|  | 4.2 | GATCCGCTTCCAGCCTTCTC | | | |  |  | GGGGAGAAGGTAAAAGCCTG | |
| ***MAPK8*** | 2 | TAGATATAGAAGACACATGTTGAGC | | | | |  | GCCCCTAATATTTAGTCTTTGA | |
|  | 3 | AACTCATGTATTTGTAGTTCCC | | | |  |  | CCAGCAGCAATCTAAGAAAA | |
|  | 5 | TTTTCTGAAGGACTTAAAGC | | | |  |  | CAACGTACTTCTGCTTAAGT | |
| ***MAPK9*** | 6 | TTGTTTTGTTCATGGCACTT | | | |  |  | GGATTTTACGACTTTGTCCT | |
|  | 8 | CCTAGCAAGTCAAGCCAGAG | | | |  |  | GCCTGGCCTCTCTATATAAA | |
| ***MAPK10*** |  | none |  | |  |  |  |  |  |
| ***MAPK11*** | 7 | CCGTGCGGAGCTTGAGAACA | | | |  |  | CCCTAAGACCTGGCATGCAG | |
|  | 10 | AGCAGCCTCCCCAGGTGTTCTT | | | | |  | CCAGCATCCTTCCAAGGAGG | |
|  | 11 | ATATCTGGCCCTGGATGTAC | | | |  |  | ATGCTCACCCTTCCACTCCT | |
| ***MAPK12*** | 3 | AGCCCGTGTCCTCTGCTGCATT | | | | |  | CCCATTGCCAACCTAGAACC | |
| ***MAPK13*** | 5 | AGCGCACTGTTACAGGTCGG | | | |  |  | CAGAGAACCCCATACCCAGG | |
| ***MAPK14*** | 4 | CACTCCAGCCTGGCAGTAGA | | | |  |  | GGGGAGAAAGGGAGAATTCA | |
| ***MAPK15*** | 1 | CGACTCAACAGTAAGGCCCC | | | |  |  | TGTGTGACGGAACAGGCCTC | |
|  | 2 | GGAGCCTCATGTCTGTAGGG | | | |  |  | ATCCTCTCCCACGCTTCTCC | |
|  | 8 | TGCCTGGGTCACACCACCTT | | | |  |  | AACTTGCAGGAAGGTGGCCCT | |
|  | 9 | ATGCCCAGGCTGTGACCTCT | | | |  |  | TTGGCAGCACAGGCAGAAGG | |
| ***NRAS*** | 3 | ATATTAGCAATTTGAGGGAC | | | |  |  | ACTTGCTATTATTGATGGCA | |
| ***PIK3CA*** | 9 | GATTGGTTCTTTCCTGTCTCTG | | | |  |  | CCACAAATATCAATTTACAACCATTG | |
|  | 9.1 | AGACTAGCTAGAGACAATGAATTAAGGG | | | | |  | CACAAATATCAATTTACAACCATTG | |
|  | 9.2 | TCTGTGAATCCAGAGGGGAAA | | | | |  | TGCTGAGATCAGCCAAATTCAGTT | |
|  | 20 | TTTGCTCCAAACTGACCAA | | | |  |  | GCATGCTGTTTAATTGTGTGG | |
|  | 20.1 | ACTGAGCAAGAGGCTTTGGA | | | |  |  | TTTGGACTTAAGGCATAACATGAA | |
| ***RAF1*** | 15 | CAGCCTGGCCCCTAGATGTCTGTGA | | | | |  | TGGGTCCTTTCGCACCAGCA | |
| ***RPS6KB1*** | 4 | AGTATGCCTGACATAGTTTG | | | |  |  | TAGGCCAAAATGTTACAACA | |
|  | 6 | AGAAGGAAAGCACAAACTCT | | | |  |  | TTACTGAATCATGTGGCTGC | |
|  | 7 | AAATCACTTTTGTTTCCAGC | | | |  |  | GGGAGGAAATATACAAAATAGAGAG | |
|  | 8 | TTATGTGACATGTTCAAACACTGC | | | | |  | GGTACAGAAAACAGATACTATTCCT | |
|  | 9 | GTTTTCTGTACCCTCATTGA | | | |  |  | CTTTGAAAACTCCACTGGTA | |
|  | 13 | TTATAGTTAACCCCCGTGAAAAGAG | | | | |  | TTGAAGAGCCCATCCTCACA | |
| ***RPS6KB2*** | 10 | GGGGAGGAGGAGGGGCAGGG | | | | |  | GGCCCTGGCTTCCCCACAGC | |
|  | 12 | AGGGGTAGGGCTGAGTCTCCAA | | | | |  | AGGTTCTCCACTTGCCACCC | |
|  | 14 | CCCCAGGGCTTCACATACGT | | | |  |  | GCAACCGAAGCCAACACTGG | |
|  |  |  |  | |  |  |  |  |  |
| **Supplemental Table S2. List of primers used to verify putative variants.** | | | | | | | | |  |
|  |  | | | | | | |  |  |
