# Supplementary material for: Mutational Analysis of EGFR and Related Signaling Pathway Genes in Lung Adenocarcinomas Identifies a Novel Somatic Kinase Domain Mutation in FGFR4
Source: PLoS One. 2007 May 9;2(5):e426. doi: 10.1371/journal.pone.0000426 (PMC1855985; doi:10.1371/journal.pone.0000426)
Supplement: Table S3 — Clinical characteristics of patients whose tumors were analyzed. Group 1 was used for sequencing the “core” genes. Group 2 was used for sequencing the “exploratory” genes. Some tumors and genes overlapped between the two groups. Smoking history is defined as never smokers (<100 lifetime cigarettes), former smokers (quit ≥1 year prior to diagnosis), or current (quit <1 year prior to diagnosis). See text and Figure 1 for more detail. 1Adeno includes adenocarcinoma with bronchioalveolar features (n = 79, n = 27 for Group 1 and 2, respectively). Abbreviations: Adeno, adenocarcinoma; SCC, squamous cell carcinoma. (0.04 MB DOC) [file pone.0000426.s003.doc]

|  | **Group 1 (Genomic DNA)** | **Group 2 (WGA DNA)** |
| --- | --- | --- |
| **# Tumors** | 217 | 93 |
| **Sex** |  |  |
| Male | 94 (43%) | 44 (47%) |
| Female | 123 (57%) | 49 (53%) |
|  |  |  |
| **Age** | 66 (39-82) | 65 (44-81) |
| **Smoking** |  |  |
| Former | 161 (74%) | 71 (76%) |
| Current | 38 (18%) | 13 (14%) |
| Never | 18 (8%) | 9 (10%) |
| **Pathology** |  |  |
| Adeno^1^ | 192 (88%) | 89 (96%) |
| SCC | 25 (12%) | 4 (4%) |
| **Stage** |  |  |
| I | 151 (69%) | 62 (67%) |
| II | 22 (10%) | 10 (11%) |
| III | 36 (17%) | 17 (18%) |
| IV | 8 (4%) | 4 (4%) |

**Supplemental Table S3. Clinical characteristics of patients whose tumors were analyzed.** Group 1 was used for sequencing the “core” genes. Group 2 was used for sequencing the “exploratory” genes. Some tumors and genes overlapped between the two groups. Smoking history is defined as never smokers (<100 lifetime cigarettes), former smokers (quit ≥1 year prior to diagnosis), or current (quit <1 year prior to diagnosis). See text and **Figure 1** for more detail. ^1^Adeno includes adenocarcinoma with bronchioalveolar features (n=79, n=27 for Group 1 and 2, respectively). Abbreviations: Adeno, adenocarcinoma; SCC, squamous cell carcinoma**.**
