# Supplementary material for: Mutational Analysis of EGFR and Related Signaling Pathway Genes in Lung Adenocarcinomas Identifies a Novel Somatic Kinase Domain Mutation in FGFR4
Source: PLoS One. 2007 May 9;2(5):e426. doi: 10.1371/journal.pone.0000426 (PMC1855985; doi:10.1371/journal.pone.0000426)
Supplement: Table S4 — List of variants verified. Group headings correspond to groups in bottom row of Figure 2. Variants found in normal tissue did not have an existing entry in dbSNP. 1A total of 5 EGFR mutations (exon 19 del, n = 1: exon 21 L858R, n = 4) have been previously reported (5). 2Variants with high frequency were not verified in all samples. If a variant was also found in DNA from five matched normals, no further samples were verified. Abbreviations: del, deletion; dup, duplication. (0.08 MB DOC) [file pone.0000426.s004.doc]

**Kinase Genes:** Somatic Mutations

| **# Tumors** | **Gene** | **Exon** | **Variant** | **AA change** |
| --- | --- | --- | --- | --- |
| 4^1^ | *EGFR* | 19 | c.2236-2250del | p.Glu746-Ala750del |
| 2 | *EGFR* | 19 | c.2239-2256del | p.Leu747-Ser752del |
| 1 | *EGFR* | 20 | c.2303_2311dup | p.Ser768_Asp770dup |
| 6^1^ | *EGFR* | 21 | c.2573T>G | p.Leu858Arg |
| 1 | *BRAF* | 15 | c.1799T>A | p.Val600Glu |
| 2 | *PIK3CA* | 9 | c.1633G>A | p.Glu545Lys |
| 1 | *FGFR4* | 16 | c.2041G>A | p.Glu681Lys |

**Kinase Genes: Variants found in matched normal tissue**

| **# Tumors** | **Gene** | **Exon** | **Variant** | **AA change** |
| --- | --- | --- | --- | --- |
| 1 | *ERBB3* | 18 | c.2150C>T | p.Ser717Leu |
| 1 | *ERBB3* | 21 | c.2595G>T | p.Gln865His |
| 1 | *MAPK3* | 7 | c.915C>G | p.Asp305Glu |
| 1 | *MAPK4* | 2 | c.359C>T | p.Thr120Met |
| 1 | *MAPK4* | 5 | c.1020G>A | p.Met340Ile |
| 1 | *MAPK6* | 2 | c.280G>A | p.Asp94Asn |
| 14 | *MAPK6* | 5 | c.868C>G | p.Leu290Val^2^ |
| 1 | *MAPK9* | 6 | c.688C>T | p.His230Cys |
| 6 | *MAPK11* | 10 | c.824G>A | p.Arg275His |
| 1 | *MAPK11* | 11 | c.916G>A | p.Ala306Thr |
| 5 | *MAPK12* | 3 | c.308C>T | p.Thr103Met |
| 1 | *MAPK15* | 2 | c.106G>A | p.Gly36Ser |
| 1 | *MAPK15* | 8 | c.779C>T | p.Arg260Trp |
| 6 | *MAPK15* | 9 | c.1073C>T | p.Pro358Leu |
| 1 | *MAPK15* | 9 | c.1117G>C | p.Ala373Pro |
| 1 | *MAPK15* | 9 | c.1141A>C | p.Thr381Pro |
| 2 | *FGFR4* | 13 | c.1772C>T | p.Ser591Phe |
| 1 | *RPS6KB1* | 8 | c.729del | p.del Ser243 |
| 1 | *RPS6KB2* | 5 | c.326A>G | p.Asn109Ser |
| 1 | *RPS6KB2* | 10 | c.800C>T | p.Pro267Leu |

**Kinase Genes:** Variants of unknown significance

| **# Tumors** | **Gene** | **Exon** | **Variant** | **AA change** |
| --- | --- | --- | --- | --- |
| 1 | *ERBB2* | 20 | c.2260C>T | p.Arg784Cys |
| 1 | *MAPK6* | 4 | c.784G>A | p.Val262Ile |

**RAS Genes: Somatic mutations**

| **# Tumors** | **Gene** | **Exon** | **Variant** | **AA change** |
| --- | --- | --- | --- | --- |
| 8 | *KRAS* | 2 | c.34G>T | p.Gly12Cys |
| 5 | *KRAS* | 2 | c.35G>A | p.Gly12Asp |
| 6 | *KRAS* | 2 | c.35G>T | p.Gly12Val |
| 1 | *KRAS* | 2 | c.37G>T | p.Gly13Cys |

**RAS Genes: Variant found in matched normal tissue**

| **# Tumors** | **Gene** | **Exon** | **Variant** | **AA change** |
| --- | --- | --- | --- | --- |
| 1 | *HRAS* | 6 | c.506G>A | p.Arg169Gln |

**Supplemental Table S4**. **List of variants verified.** Group headings correspond to groups in bottom row of **Figure 2**. Variants found in normal tissue did not have an existing entry in dbSNP. ^1^A total of 5 EGFR mutations (exon 19 del, n=1: exon 21 L858R, n=4) have been previously reported (5). ^2^Variants with high frequency were not verified in all samples. If a variant was also found in DNA from five matched normals, no further samples were verified. Abbreviations: del, deletion; dup, duplication.
